# Adaptation cannot keep pace with projected temperature increase

**DOI:** 10.1016/j.isci.2023.108403

**Published:** 2023-11-07

**Authors:** Shuai Chen, Jie-Sheng Tan-Soo, Hai-Jian Ye

**Affiliations:** 1 Academy of Social Governance, Zhejiang University, Zhejiang, China; 2 China Academy for Rural Development (CARD) and School of Public Affairs, Zhejiang University, Hangzhou, China; 3 Lee Kuan Yew School of Public Policy, National University of Singapore, Singapore, Singapore

**Keywords:** Earth sciences, Climatology, Environmental science, Environmental health

## Abstract

An emerging argument is that since humans can readily adapt to changing climatic conditions, there is less need to pursue aggressive emissions mitigation strategies. As temperature adaptation is a function of repeated exposure over time, we need empirical approaches that can depict individuals’ temperature history to rigorously examine this claim. Using a longitudinal dataset representative of China, we construct lifetime temperature exposure unique to each individual based on their birth-dates, birth-locations, and movement history. We show that a 1°C increase in individualized temperature anomalies cause a 2% decrease in 1 standard deviation (S.D.) of well-being, where most of the impacts are driven by “hotter-than-expected” weather. In turn, while the adverse impacts of future temperature changes wane after accommodating for adaptation, acclimatization is unlikely to keep pace with future temperature increases except in the net-zero emissions scenario, indicating that stringent greenhouse gas (GHG) emissions cuts are still needed even in this less-pessimistic scenario.

## Highlights

• Adaptation to temperature is a function of one’s previous exposure
• We use location history and birth-dates to construct temperature experience
• Projected temperature increase is faster than pace of adaptation

## Graphical abstract

**Figure:**
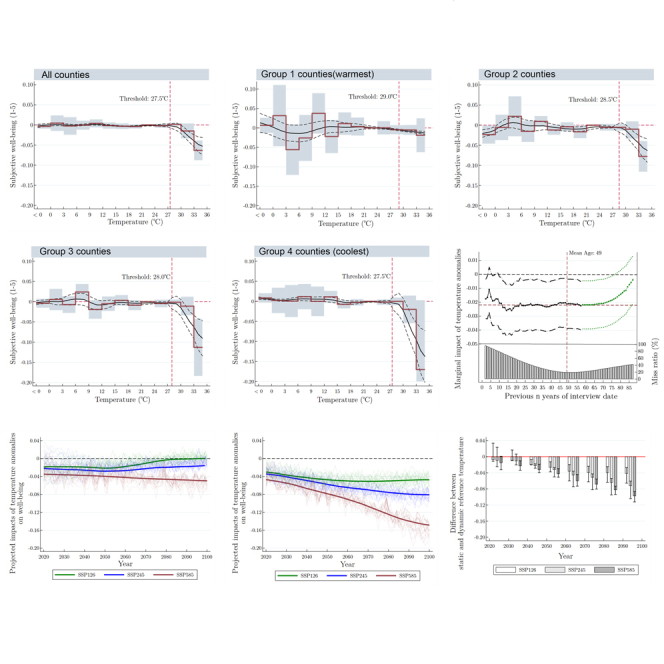

## Introduction

Even though Earth’s climate has drastically changed multiple times over its history, the current iteration differs in one key way: the existing and projected rate of temperature rise greatly outpaces that of past events’.^1^ For instance, temperature has increased by 4°C–5°C over 7,000 years since the last ice age, with 1°C in just the last 200 years^2^ In this regard, climate researchers project widespread global disasters and catastrophes as it is unlikely countries and cities can adapt in time if temperature continues to increase at current speed.^3^ There is already evidence that anomalous weather conditions can cause widespread damages. For instance, the unprecedented 2003 European heat wave was estimated to cause an excess 14,729 mortalities in France.^4^

On the other hand, it is also true that humans have shown propensity in rapidly adapting toward new climatic baselines. Using the same example from earlier, due to adaptation measures taken by the government and public, an equally intense heatwave in Europe just three years later in 2006 saw excess mortalities reduced drastically to 2,065.^5^

As such, an emerging argument is that current and planned emissions mitigation strategies are overly aggressive as humans can readily adapt to future climatic changes.^6^^,^^7^^,^^8^ While there is some merit in this claim as humans have indeed shown ability in adapting to various environmental changes, further investigation is needed to rigorously examine this contention.

To assess humans’ ability to adapt to climate change, we need to first examine the impact of climate on well-being.

Brereton et al.^9^ conducted one of the earliest studies using individual-level data, utilizing information from Ireland. Their research revealed a positive correlation between happiness and various precisely measured environmental factors. Particularly, they discovered that the minimum temperature in January and the maximum temperature in July significantly impact overall life satisfaction. In contrast, Denissen et al.^10^ came to opposite findings as the mood of their German respondents did not display any adverse impacts to temperature. One plausible explanation for these contrasting findings is that both studies made use of cross-sectional datasets, which in turn runs the risk of confounding explanations, i.e., variables that change with temperatures and also affect happiness independently.^11^

In this regard, there are several recent studies that use panel datasets to explore the relationship between temperature and well-being.

Feddersen et al.^12^ conducted an investigation into the effects of climatic conditions on subjective well-being (SWB), utilizing a comprehensive longitudinal dataset from Australia. Contrary to expectations, they found a positive correlation between the daily duration of sunlight and subjective well-being (SWB), while temperature demonstrated no significant influence.

Both Hou et al.^13^ and Hua et al.^14^ used the same longitudinal dataset to investigate the impact of temperature on mental health of Chinese residents. While both studies arrive at the same conclusions that high temperature is detrimental to psychological well-being, a key difference is that the former proposed a linear relationship between daily average temperature and mental health whereas the latter used a non-linear function via temperature bins.

Up until recently, most studies investigating the relationship between temperature and happiness have primarily relied on self-reported responses from individuals, such as subjective well-being, overall life satisfaction, or mental health. In the Economics literature, these outcomes are typically interpreted as either evaluative or experienced utility.^15^ However, there is an emerging trend in the utilization of alternative data sources, wherein researchers perform text analytics on social media posts to assign quantifiable metrics for “happiness” or sentiment.

An exemplary instance is by Baylis et al.^16^ In their study, they aggregated sentiment at the urban-daily level, and unearthed that users of prominent social platforms like *Twitter* and *Facebook* within the United States display a propensity to convey negative sentiments during periods of both high and low temperature extremes. Wang et al.^17^ derived analogous findings through their analysis of posts from *Weibo*, which is recognized as the predominant social media platform in China. Additionally, Baylis Baylis^18^ expanded the scope of the *Twitter* dataset to incorporate an additional six countries. By utilizing temperature bins as the explanatory variable, an inverted U-shaped relationship between temperature and negative sentiments was discerned.

While these studies mostly confirm the adverse impacts high temperature has on human well-being, a shortcoming of this literature is that most fail to account for individual climate adaptation which restricts their usage in conducting long-term impact projections.

On this note, there is a strand of literature in climate change science devoted toward uncovering how adaptation can mediate the detrimental effects of climate change.

One of the first ways used to estimate climate adaptation is via long-difference.^19^ The intuition behind this approach is that a panel dataset, if of sufficiently long time span, can be separated into two sets of equal periods. Differences in their respective coefficients or marginal estimates can thus be interpreted as evidence of climate adaptation. While this approach has been widely applied in settings such as agriculture,^19^^,^^20^ household water usage,^21^ and migration,^22^ its hefty demand on datasets precludes wider usage.

Second, Medina-Ramon and Schwartz^23^ made use of existing climatic differences between US cities to show that the temperature-mortality relationship is highly dependent on location’s average temperature where places that are cooler are more susceptible to anomalously warm days.

Third and related, the previous approach can be used to project future outcomes by extrapolating the temperature-outcome relationship from say, currently hot places to “predicted-to-be” hot places.^24^^,^^25^

The central assumption behind all these existing methods is that climate adaptation is a function of prolonged exposure. The long-difference approach assesses climate adaptation by comparing long-term and current responses over a study period. While it offers a broad view of adaptation regardless of specific mechanisms, and can be applied to any past period with available data, it falls short when used for future predictions. This is because it assumes human climate adaptation remains constant over time, which may not be the case. In practice, results can vary widely when computing adaptation over the past thirty versus say, fifty years. In essence, while the long-difference approach effectively portrays historical human climate adaptation, it is not directly suited for forecasting future adaptation.

The extrapolation method employed by Auffhammer^25^ and Heutel et al.^24^ is a valuable extension, as they leverage on existing temperature-outcome profile disparities. Their main insight is to use spatial differences in these profiles to describe how human climate adaptation evolves over time. Essentially, this method extrapolates how currently cold regions might respond to rising temperatures in the future based on the response of currently warm regions. While this approach holds promise in projecting future adaptations to climate change, it overlooks a critical issue: whether the adaptation of residents in currently warm regions can accurately predict the future responses of those in currently cold regions. In summary, the extrapolation technique relies on strong assumptions to predict adaptation, and future research should work toward relaxing these or better capturing individual differences and experiences.

In this regard, it stands to reason that the optimal way to assess one’s level of adaptation is to compute the climatic conditions that one has experienced over their lifetime. However, such empirical strategy has yet to be implemented due to lack of information that can accurately depict one’s lifetime experience. This is another knowledge gap we attempt to address in this study.

In this regard, the central goal of this study is to weigh in on this important debate of whether humans’ adaptation to changing temperature can catch up with climate change. To do so, we use a longitudinal dataset representative of China where we carefully trace out each person’s unique and dynamically changing historical reference temperature by using information on birth-dates, locational history, and survey dates.

Our empirical results suggest that a 1°C increase in personalized temperature anomalies results in a 2% diminution in one standard deviation of individual well-being, where most of the impacts are driven by “hotter-than-expected” weather. When projected across different emissions pathways, we show that even though adaptation can indeed mediate or decelerate the detrimental impacts of climate change, the former is slow compared to the rate at which temperature changes. Importantly, humans’ responses and adaptations can only catch up with climate change under the SSP126 or net-zero emissions scenario.

This study advances from the current literature in the following ways.

First, existing longitudinal studies mostly rely on a combination of individual-level fixed-effects with temperature to recover the impact of temperature on well-being.^13^^,^^14^ Essentially, this approach assigns all individuals from the same region to the same historical reference temperature. However, it is clear from existing evidence that adaptation is dependent on personal experience, and prolonged exposure.^26^^,^^27^^,^^28^ Similarly, some adaptation measures are location-specific, which is also dependent on prolonged exposure to the underlying climatic conditions. As such, our novel approach of using individuals that lived in the same location since birth, and using their age to construct personalized historical reference temperature can precisely account for both individual and regional climate adaptation.

Second and related, other than using one’s locational history and age, we also rely on exogenous variation in survey dates to form historical reference temperature. The rationale is that one’s expectations of the weather are highly dependent on the time of the year. In this regard, these three sources of variation provide us with unique temperature anomalies for each individual at each point in time.

Third, a crucial contribution of this study is that instead of extrapolating statistical relationships across time and space, we use one’s actual experiences to account for climate adaptation. This approach allows us to dynamically update one’s climate adaptation in a way that mimics the actual speed at which individuals acclimatize in reality.

## Results

### Regional and temporal heterogeneity in temperature and well-being

We initiate our investigation into the connection between temperature and well-being by employing temperature bins as the principal variables in the estimation of Equation 1.

**Figure 1:**
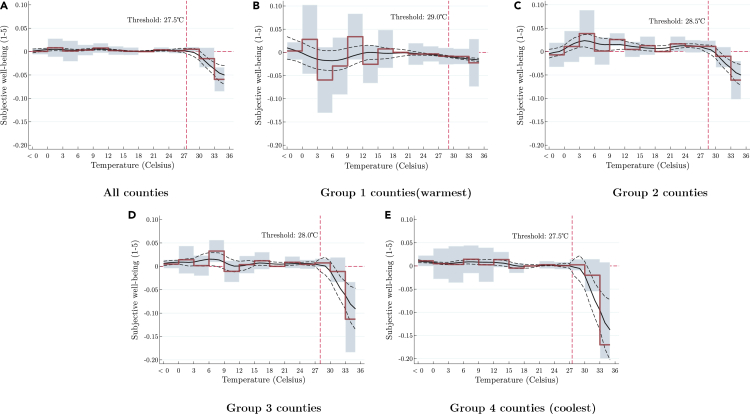
Subjective well-being and temperature bin across different parts of China The graphical illustrations within this investigation showcase the determined coefficients of temperature bins, which divide the tally of days in the antecedent 30-day span into separate 3°C segments, and their ensuing impact on subjective well-being. Each derived coefficient is meant to be interpreted in contrast to the reference group nestled within the 18°C–21°C temperature ambit. The dotted trajectories plotted on each graph symbolize the 95% confidence boundaries that correspond to the estimated coefficients, whereas the vertical demarcation indicates the temperature degree at which the inflection point takes place.

Panel A of Figure 1 showcases the coefficients corresponding to each temperature bin for the comprehensive sample. Our empirical outcomes align with those of preceding research that explored the impacts of temperature on human well-being, thereby endorsing the conclusion that higher temperature indeed exerts a detrimental influence on subjective well-being (SWB). Relative to the reference temperature range of 18°C–21°C, SWB is observed to decrease by up to 0.05 for each successive day where the mean temperature surpasses 27.5°C.

**Figure 2:**
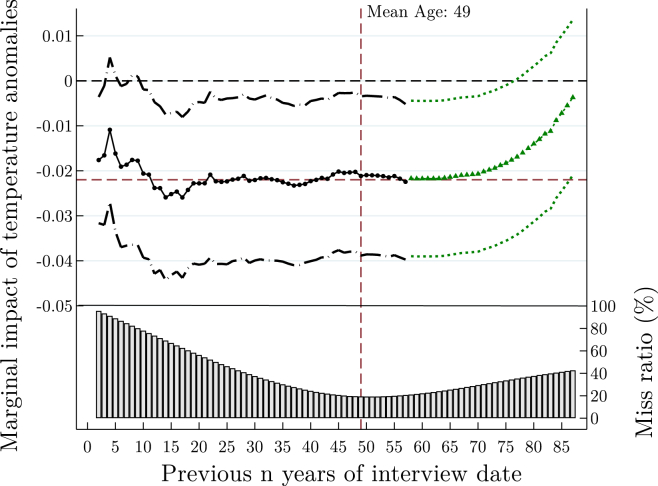
Formation of reference temperature at different ages The figure plots the coefficients collected from different constructions of historical reference temperature. The labels in the *x*-axis represent the last *n* years over which historical reference temperature is averaged over. The dots in the top half of the figure show the corresponding coefficients for temperature anomalies, and are joined by lines. The 95% confidence intervals are represented by dashed lines. The green portions are based on extrapolations as we do not have temperature dating before 1950s. The bottom half of the figure shows the proportion of lifetime years’ missed based on each choice of preceding years.

Considering the vast geographical span of China which encompass diverse climatic zones, we hypothesize that uniform heat tolerance may not be applicable across the entire population. For instance, inhabitants of hotter climatic zones might exhibit greater heat tolerance. To explore this hypothesis, we segment the country into four zones based on the average annual temperature at the county level over the preceding 50 years, and subsequently repeat the estimation for each group. Panels B through E of Figure 2 depict the graphical representation for each county set, organized in descending order of average annual temperature.

Firstly, a conspicuous trend manifests across the four groups: the temperature threshold at which SWB begins to be impacted diminishes as we traverse from warmer to cooler regions of China. Precisely, the temperature inflection point for inhabitants in Group 1 (the warmest) counties initiates at around 29°C, whereas it commences at 27°C for those residing in Group 4 (the coolest) counties.

Secondly, the magnitude of the impact illustrates a gradient alteration in sync with the average annual temperature. Inhabitants from Group 1, while demonstrating a higher temperature inflection point, also bear the most modest impact, evidenced by a coefficient of −0.02 at the most elevated temperature bin. Conversely, the SWB of residents from Group 4 (the coldest counties) declines by approximately −0.17 at the peak temperature bin.

Beyond classifying counties according to their annual temperature, we delve into stratifications anchored on geographical location. Figure S3 delineates a series of graphs for the northern, southern, eastern, western, and central territories of China. We discern disparate patterns in the temperature-SWB trajectories across these distinctive regions.

Other than regional heterogeneity, our dataset can also be used to investigate temporal variation in temperature-well-being profile. To do so, we estimate Equation 1 for each China Family Panel Survey (CFPS) wave. The three graphs in Figure S4 show a consistent trend where the impact of high temperature days on SWB gradually wanes as we advance from 2010 to 2018 survey wave.

In all, these three set of analyses confirm that adaptation to underlying climatic conditions exist not only at the regional level, but is also a dynamic process.

### Individualized temperature anomalies and well-being

The preceding findings have established that while high temperatures adversely affect well-being, the definition of “high” and the level of discomfort caused by heat differ significantly across locations, rather than being uniform throughout the country and time. To gain a more nuanced understanding of the relationship between climate change and well-being, we utilize an alternative metric, temperature anomalies, which denotes the difference between current and historical temperatures. While prior studies have used temperature anomalies in their empirical approaches,^28^^,^^29^^,^^30^ we differ in how we construct this covariate.

Due to the lack of personal information, earlier studies mostly used identical temperature anomalies for all observations made during the same time period. However, it is plausible that individuals have varying expectations about temperature based on their unique experiences. To address this, we leverage the rich information inherent in our dataset, defining historical temperature as the average temperature in the same county over the identical 30-day span commencing from the respondent’s birth year. For instance, if a respondent’s interview falls on August 15^th^, their current temperature is quantified as the average temperature spanning from July 14^th^ to August 15^th^. Conversely, their reference historical temperature is established as the average temperature for this identical 30-day duration starting from their birth year and extending up to the year immediately preceding the survey execution.

Table 1 furnishes our primary regression results, where we incrementally incorporate fixed effects and diverse covariates. We inaugurate the process with the estimation of a cross-sectional OLS model, devoid of fixed effects or controls, to evaluate whether temperature anomalies manifest any noticeable correlation with SWB. The coefficient for temperature anomalies featured in Column (1) is positive and statistically non-significant. In the subsequent stages, we incorporate survey date and individual-level fixed effects, whereupon we note the coefficient for temperature anomalies to be negative and statistically significant. The contrast in outcomes between Columns (1) and (2) underlines two pivotal facets of our empirical methodology.

**Table 1:** Baseline results

*Dep. Var.*	Subjective well-being (level 1–5)			
	(1)	(2)	(3)	(4)
Temperature-anomalies-birth	0.0189 (0.0248)	−0.0226∗∗∗ (0.0084)	−0.0226∗∗ (0.0088)	−0.0227∗∗∗ (0.0087)
Income				0.0158∗ (0.0087)
Age				0.0204 (0.0256)
Marital status				0.1650∗∗∗ (0.0334)
Educational level				0.0091∗ (0.0051)
Employment status				0.0196 (0.0234)
Feeling of discomfort in last two weeks				−0.0920∗∗∗ (0.0191)
Wind speed-birth			−0.2229 (0.1436)	−0.2239 (0.1424)
Humidity-birth			0.0071 (0.0252)	0.0062 (0.0248)
Precipitation-birth			−0.0001 (0.0012)	−0.0001 (0.0012)
Solar duration-birth			−0.0015 (0.0029)	−0.0011 (0.0028)
Date *FE*	No	Yes	Yes	Yes
Individual *FE*	No	Yes	Yes	Yes

*Notes*: The total number of observations is 26,583. All models are estimated using survey weights. Additionally, Column (4) includes second-order weather polynomials, although they are not explicitly displayed in this section. Standard errors are provided in parentheses and clustered using two-way clustering at both the county and date levels. The significance levels are indicated as follows: ∗∗∗p < 0.01, ∗∗p < 0.05, ∗p < 0.1.

Firstly, when engaging a subjectively defined outcome, it becomes indispensable to execute within-individual analyses to preclude the impacts of idiosyncratic variables on the dependent factor.

Secondly, as explained in Hsiang,^11^ incorporating unit and period fixed effects is necessary to remove temperature’s remaining endogeneity.

As the coefficient for temperature anomalies demonstrates comparable stability across the residual fixed-effects models featured in Columns (3) and (4), our analysis primarily focuses on the optimal specification delineated in Column (4). This preferred model incorporates comprehensive fixed effects, weather, and individual control variables.

The signs and statistical significance of the control variables align with expectations and findings from existing literature. Individuals with higher income and education levels tend to report higher levels of SWB. We also include a control variable indicating whether the individual experienced any discomfort in the last two weeks to account for any incidental negative ailments affecting SWB. As expected, the coefficient for this variable is strongly negatively correlated with SWB. Besides personal characteristics, we incorporate various weather-related covariates since temperature is correlated with other contemporary weather phenomena.

Even after accounting for all these controls, we discover that the coefficient for temperature anomalies continues to maintain a negative correlation with SWB. Specifically, the estimated influence of temperature anomalies on SWB is −0.023, implying that for every 1°C escalation in which the actual temperature surpasses the reference temperature, self-reported happiness diminishes by −0.023. This corresponds to approximately 2% of one standard deviation of SWB.

While temperature anomalies, on balance, lean toward the positive end (signifying an aggregate increase in temperature over time in China), they span from −2.5°C to 3.7°C. Here, positive values denote temperatures exceeding the historical average, and the inverse is true for negative values (refer to Figure S5 for the histogram of temperature anomalies). Therefore, previously established results infer that SWB declines in the face of positive temperature anomalies (i.e., weather hotter than anticipated) and ascends amid negative temperature anomalies (i.e., weather colder than anticipated).

We delve further to explore whether our baseline findings are primarily attributable to positive or negative anomalies, or equally impacted by both. To accomplish this, we substitute the observations of negative anomalies with zeros, our objective being to single out the influences of positive anomalies on SWB.

Column (1) of Table 2 reveals that the coefficient for positive temperature anomalies is approximately 70% larger than the baseline, registered at −0.036. This underscores that the earlier observed effects are primarily driven by higher-than-anticipated temperatures. We replicate this analysis by substituting positive anomaly observations with zeros. The coefficient for negative temperature anomalies, while remaining negative (indicating incremental effects on SWB), is minor and statistically insignificant (refer to Table 2, Column (3)). Lastly, analogous outcomes are discerned when both transformed variables are incorporated in the same regression model (Table 2, Column (4)). These results corroborate that, relative to lower-than-expected weather, positive temperature anomalies exert a more pronounced influence on SWB. Consequently, the baseline findings can be construed as signifying that warmer-than-anticipated weather predominantly fuels the association between temperature anomalies and SWB.

**Table 2:** Isolating different effects of temperature anomalies

*Dep. Var.*	Subjective well-being (level 1–5)			
	(1)	(2)	(3)	(4)
Abs (Temperature-anomalies-birth)	−0.0363∗∗∗ (0.0139)			
Positive values of temperature-anomalies-birth (“hotter-than-expected”)		−0.0362∗∗∗ (0.0130)		−0.0391∗∗∗ (0.0142)
Negative values of temperature-anomalies-birth (“colder-than-expected”)			−0.0061 (0.0217)	−0.0155 (0.0231)
Mean [SD] of Temperature-anomalies	0.91 [0.65]	0.73 [0.72]	−0.17 [0.40]	–

*Notes*: The total number of observations is 26,583. All models are estimated using survey weights and incorporate an extensive range of covariates, including survey dates fixed-effects (FE), individual-level fixed-effects (FE), weather controls, and individual controls. The weather controls encompass relative humidity, wind speed, hours of sunlight, and precipitation, with second-order polynomials considered. Individual controls consist of per-capita household income, age, marital status, educational level, and self-reported discomfort experienced within the past two weeks. Standard errors are provided in parentheses and clustered using two-way clustering at both the county and date levels. The significance levels are indicated as follows: ∗∗∗p < 0.01, ∗∗p < 0.05, ∗p < 0.1.

### Estimates without individualized information

Next, we consider how our estimates would change if we do not have individualized information, and instead rely on fixed historical period to construct temperature anomalies.^17^^,^^29^^,^^30^ The results in Column (2) of Table 3 show that by using temperatures from 1980 to 2000 to form historical reference temperature, the impact of temperature anomalies is around 22% larger in magnitude at −0.028. Similar outcomes are observed if we used temperature from 1960 to 1980 instead. The most plausible explanation is that because fixed-period historical reference temperature did not take recent experiences into consideration. This is especially true as global (and China’s) temperatures have increased considerably in recent decades. As such, temperature anomalies constructed using non-updated reference temperatures yield more and larger positive temperature anomalies, and eventually amplify its’ impact on SWB.

**Table 3:** Using fixed time period as reference temperature

*Dep*. *Var*.	Subjective well-being (level 1–5)		
	(1)	(2)	(3)
Temperature-anomalies-birth	−0.0227∗∗∗ (0.0087)		
Temperature-anomalies-Fix 1980-2000		−0.0277∗∗∗ (0.0081)	
Temperature-anomalies-Fix 1960-1980			−0.0284∗∗∗ (0.0081)
Date *FE*	Yes	Yes	Yes
Individual *FE*	Yes	Yes	Yes
Weather controls	Yes	Yes	Yes
Individual controls	Yes	Yes	Yes
Mean [SD] of Temperature-anomalies	0.62 [1.18]	0.73 [1.12]	0.75 [1.13]

*Notes*: The total number of observations is 26,583. All models are estimated using survey weights and incorporate an extensive range of covariates, including survey dates fixed-effects (FE), individual-level fixed-effects (FE), weather controls, and individual controls. The weather controls encompass relative humidity, wind speed, hours of sunlight, and precipitation, with second-order polynomials considered. Individual controls consist of per-capita household income, age, marital status, educational level, and self-reported discomfort experienced within the past two weeks. Standard errors are provided in parentheses and clustered using two-way clustering at both the county and date levels. The significance levels are indicated as follows: ∗∗∗p < 0.01, ∗∗p < 0.05, ∗p < 0.1.

On the other hand, even in the absence of individualized information, it is still possible to update reference temperature to most recent years. In Figure 2, we use average temperature from *n* preceding years to form references, and graph their respective coefficients. The drawback of this approach is that we are unable to form historical reference temperature up until individuals’ birth years. As such, using the vantage point of our dataset, we compute the proportion of years missed for each number of preceding years (assuming we use 25 preceding years to form reference temperature. For a 50-year-old individual, we would have missed out 25 years of his earlier life, thus generating years-missed rate of 50%. On the other, we would have included five additional years for a 20-year-old individual. In this case, the years-missed rate is 20% (from (25-20)/25)).

The first insight from Figure 2 is that the coefficients for temperature anomalies are statistically insignificant if we use average temperature from 2 to 10 preceding years to form reference temperature. The reason is found in the high proportion of lifetime years missed, at around 87%.

Second and related, this approach subsequently yields coefficients closer to the baseline estimate as we include more preceding years, thereby reducing proportion of missed years. However, it should be emphasized that it is possible to over-include number of preceding years. The right-hand-side of Figure 2 shows that the coefficients will again diverge from the baseline estimate, and become statistically insignificant as more preceding years are included.

In all, these two counterfactual analyses highlight the main advantages of our approach. First, we will likely overestimate impact of temperature anomalies on SWB if fixed time period is used to construct historical reference temperature. This is because latest experience to increasing temperature is not taken into account. Second, the analysis will likely suffer from attenuation bias if we simply use the same number of preceding years for everyone.

### Formation of historical reference temperature

When constructing individualized temperature anomalies, the definition of “historical reference temperature” is a key consideration. Although there are many ways in which historical temperature can be formed, we have so far constructed it by using only (1) individuals where we are fully certain of their locational history, and (2) temperature averaged over their entire lifetime.

In this sub-section, we relax these restrictions in two ways.

First, we expand the sample to include more respondents.

We start with the broadest sample, and subsequently filtering by including more location restrictions. In doing so, we can examine how the marginal impacts of temperature anomalies change as we introduce more precise computations of historical reference temperature.

We first begin with the full sample of respondents from CFPS. Next, we use only respondents that did not change location throughout the three survey waves. Third, we filter the sample by using only respondents that retain same location in the three survey waves, and also reported same location as birth-place at 12 years old. In the following iterations, we add the condition of location at three years old, and so on.

As we proceed from a broad sample where individuals’ location is not entirely accounted for to a narrow sample where we are almost certain of their lifetime locations, we discern a general increasing trend for temperature anomalies coefficients (Table S3).

That is, temperature anomalies have a larger marginal impact on SWB as we are more certain of the respondents’ location history. This trend is most likely due to increasing precision of historical reference temperature, and thus lessening the risk of attenuation bias.

Up until now, one limitation of our sample is that we used respondents that did not change location. In here, we make use of additional information from CFPS to construct historical reference temperature by using temperature from multiple locations for the same respondent. Specifically, respondents who had different locations at three and twelve years old from birth will also report their exact location at then. As such, we can now construct historical reference temperature for respondents that were at different locations at various points in time. One limitation of this approach is that CFPS do not fully reveal respondents’ government-defined location codes. As such, we are only able to identify new locations for a small number of such respondents. The findings in Column (7) of Table S3 resemble the baseline results, indicating that our conclusions are not influenced by sample selection. Additionally, we are able to replicate all results from this study using the sample in Column (2) and Column (7) of Table S4 (results are available upon request).

The second way in which we alter the construction of historical reference temperature is temporally where we examine if reference temperatures are more formed by early life’s or more recent experiences. This is an important distinction as global temperature has steadily increased over the past 40 years at the rate of 0.2°C/decade. As such, knowing how temperature expectations are formed would allow us to project climate change impacts more accurately.

We begin by using only temperature (for the same 30-day period prior to interview date *t*) from birth year (i.e., *age* = 1) and cumulatively include older ages up until 40. When constructed in this manner, historical temperature relies more on latter-life experiences as *age* gets larger. To interpret the results, each coefficient is plotted against their corresponding *age* in Figure S6. We see that the marginal impacts of temperature anomalies on SWB broadly resemble a U-shaped relationship. Impact of temperature anomalies on SWB increases in magnitude as more years (or latter-life experiences) are included. The largest marginal effect is at around ages of 24–26 years old. Following that, we observe a gradual decrease in magnitude with more years added, and turn statistically insignificant at around 32 years.

We can further contextualize these findings by compartmentalizing ages into typical milestones of human lifespan—infant, childhood, teenager, young adult, and adult. The results in Table S4 are largely consistent with the earlier ones as we see that anomalies from temperature formed during early adulthood (around 18 to 40 years old) have the largest impact on SWB. Conversely, temperatures experienced past the age of 40 have a diminished role in forming reference temperature.

### Robustness checks

To validate the integrity of our baseline results and ensure they are not subject to the influences of sample selection or modeling assumptions, we administer the following robustness checks: (1) the utilization of a population sample born post-1951, (2) the adoption of wet-bulb and maximum temperatures in place of average temperatures, (3) using other expressions of SWB, (4) using day-measured temperature anomalies, (5) using time-frames other than one-month, and (6) various ways of clustering standard errors.

Our baseline findings do not deviate from the robustness analyses. Full details of these robustness analyses are presented in the STAR Methods section.

### Climate change projections

A key difference between our empirical approach and that of earlier studies is that the relationship between SWB and temperature is dynamic. For instance, in a temperature bins model, the impact of a 30°C-day on SWB remains the same regardless of year. However, the same 30°C-day can have different impacts in our temperature anomalies model at various time periods, depending on earlier temperature conditions.

In this sub-section, we build on this dynamic relationship to examine how temperature anomalies will affect SWB under different GHG emissions mitigation scenarios.

**Figure 3:**
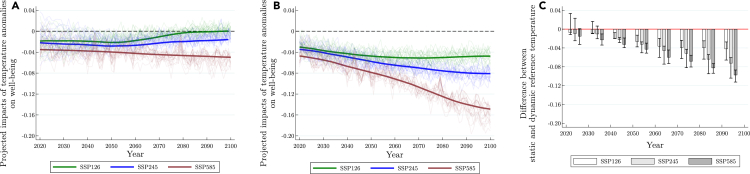
Projected impacts of temperature on subjective well-being under different computations of historical reference temperature These graphs show the projected impacts of temperature on subjective well-being by computing temperature anomalies under (A) continuously updating according to age, and (B) fixed reference period of 1980–2000. The three emissions scenarios are chosen to represent (1) net-zero emissions (SSP126), (2) middle-of-road (SSP245), and (3) very high emissions (SSP585). Faint lines indicate province-level temperature projections. (C) shows the difference in temperature anomalies between (A) and (B) at each decade (summary statistics is shown in Table S12). The faint lines represent different climate models (a detailed list of selected models in Table S11). We have additionally re-drawn (A) using different projection of average age (see Figure S7).

Under this backdrop, the projected impacts of temperature anomalies on SWB are shown in Figure 3. We make the following observations.

First, across all three scenarios, compared to continuously updated historical reference temperature, the impact of temperature anomalies on SWB is always higher if historical reference temperature is fixed. This is expected as global temperature is projected to increase regardless of mitigation scenarios. As such, if we assume that individuals do not update their reference temperature, they will inadvertently be faced with increasing temperature anomalies (and thus larger adverse impacts to SWB) over time.

Second and more importantly, even if reference temperature is continually updated (i.e., climate adaptation is considered), society still incur negative impacts on their SWB as the speed of temperature increase is outpacing the rate of acclimatization. An important point of departure is that under the net-zero emissions scenario (SSP 126), temperature anomalies will no longer affect SWB at around year 2080 as that is when the speed of temperature increase has slowed down sufficiently for individuals to fully adapt. On the other hand, we do not observe similar outcomes under the business-as-usual and in-between mitigation scenarios.

In the regard, the main takeaway is that even though individuals can adapt to rising temperature over time, this does not negate the need for pursue more aggressive emissions mitigation measures as the rate of temperature increases would otherwise outpace adaptation.^6^^,^^7^^,^^8^

### Conclusion

Climate projections mostly arrive at a gloomy future unless GHG emissions are drastically reduced. However, it is also true that humans have shown ability to adapt to changing climatic conditions. As such, the debate of whether humans can adapt fast enough in response to climate change requires a thorough empirical investigation.

This study contributes to this endeavor by examining the relationship between temperature anomalies and well-being. Specifically, we construct each person’s lifetime temperature exposure by using information on their location history, birth-dates, and interview dates. In turn, this approach allows us to use one’s actual experiences to project future climate adaptation, instead of extrapolating statistical relationships across time and space.

We first establish empirically that temperature adaptation is influenced by underlying climatic conditions and repeated exposure over time. Therefore, we first confirm empirically that temperature adaptation is a function of underlying climatic conditions, and repeated exposure over time. Our analysis underscores that while elevated temperatures exhibit a negative correlation with SWB, the ability to endure heat exhibits substantial variations across diverse climatic zones and temporal periods. Consequently, the adoption of individual-specific temperature anomalies, conceptualized as the difference between the actual and the reference temperatures, facilitates a more dynamic modeling of the relationship between temperature and SWB. This approach allows for the incorporation of temperature adaptation factors. After integrating an array of control variables and fixed-effects into the model, our results suggest that a 1°C rise in temperature anomalies causes a 0.023 decrease in SWB, which equates to roughly 2% of a single standard deviation.

Second, as our model and findings suggest that individuals can adapt to higher temperature over time, it is thus important to examine if this implies that global warming will be less detrimental to well-being over time as suggested by “climate dismissers”, and negates the need to reduce emissions.^6^^,^^7^^,^^8^ We find that even under constantly updated reference temperature, there still will be adverse impacts on SWB as temperature increases outpace acclimatization. The only exception is in the net-zero emissions scenario (SSP126) where Chinese residents eventually fully acclimatize at around year 2075.

## Discussion

Our findings provide the following research and policy implications.

First, due to humans’ ability to adapt to underlying and changing climatic conditions, individualized temperature anomalies can better represent the relationship between climate change and human well-being. Our findings on the formation of historical temperature also provide guidance for future research using temperature anomalies. Up until now, historical or reference temperature have been defined in highly varied ways across studies. In this study, we carefully construct individualized historical reference temperature using information on location and birth-dates. Our findings suggest that, in the absence of individualized information, future studies using temperature anomalies to estimate impacts of climate change on human outcomes should always use constantly updated reference temperature that can best match up to average population age to form historical reference temperature. In all, this study is part of a small, but growing literature that is taking a closer look at how we can better empirically model the relationship between temperature and various climate-related outcomes. It is likely that there are more intricate relationships between temperature and human outcomes that can be uncovered by blending rigorous statistical modeling with better understanding of humans’ behaviors.^31^

Second, the history of climate change science is filled with controversies and skepticism.^32^^,^^33^ In turn, one of the latest arguments made against mitigating GHG emissions is that we can easily adapt to changing climatic conditions, and that current commitments toward emissions mitigation are over-zealous.^6^^,^^7^^,^^8^ This argument is not entirely unmerited as humans have shown propensity to adapt, and some studies even projected climate change to reduce mortality following adaptation.^24^ With respect to temperature changes, we project that climate change will continue to negatively affect well-being as humans cannot adjust in time to rising temperature. The only exception is for the net-zero emissions (SSP126) scenario where temperature impacts will decay to negligible at around year 2080. Consequently, our results debunk the assertion that humans can adequately adapt in time to rapidly changing temperatures, and underscore the immediate need to implement rigorous emissions mitigation policies.

### Limitations of the study

This study is not without limitations. First, while our approach of constructing individualized temperature anomalies is novel, it comes at a cost of relatively heavy data requirement where knowledge on the locational history of every individual is needed. Due to lack of complete personal information, we fulfill this condition by only limiting to respondents that did not move in their lifetime. The tradeoff is that our findings may not be generalizable to the subset of population that chooses to move. As higher-quality datasets emerge, it is likely that one’s location history is more readily available, and future studies in this area can expand to include the entire population. Second, even though we use subjective well-being as an outcome that is encompassing of one’s general well-being, one can rightfully doubt its objectiveness and comparability between individuals. On this note, temperature anomalies should negatively affect various aspects of human outcomes, and future studies can readily apply our methods to other geographic settings and expressions of well-being.

## STAR★Methods

### Key resources table

**Table:** 

REAGENT or RESOURCE	SOURCE	IDENTIFIER
**Deposited data**		

Individual well-being data	China Family Panel Survey	https://opendata.pku.edu.cn/dataverse/CFPS?language=en
Historical daily weather data	China Meteorological Data Service Center	http://data.cma.cn/
Climate projection data	the NEX-GDDP-CMIP6 dataset	https://www.nccs.nasa.gov/services/data-collections/land-based-products/nex-gddp-cmip6
Age projection data	United Nations World Population Prospects	https://population.un.org/wpp/

**Software and algorithms**		

Stata	Stata 16	https://www.stata.com/new-in-stata/
Python	Python 3.7.6	https://www.python.org/downloads/release/python-376/
ArcGIS	ArcGIS 10.8	https://www.esri.com/en-us/home

### Resource availability

#### Lead contact

Further information and requests for resources should be directed to and will be fulfilled by the lead contact, Jie-Sheng Tan-Soo (jiesheng.tan@nus.edu.sg).

#### Materials availability

The study did not generate new materials.

#### Data and code availability

• Data: Individual well-being data were obtained from the China Family Panel Survey (CFPS), accessible through the Peking University Open Research Data Platform at https://opendata.pku.edu.cn/dataverse/CFPS?language=en. Daily weather information data were sourced from the China Meteorological Data Service Center, available at http://data.cma.cn/. Climate projection data were obtained from the NEX-GDDP-CMIP6 dataset, accessible at https://www.nccs.nasa.gov/services/data-collections/land-based-products/nex-gddp-cmip6. Age projections are obtained from United Nations World Population Prospects at https://population.un.org/wpp/. All data and models underwent processing using Stata 16.0, Python, and ArcGIS. Figures were generated using Stata 16.0 and ArcGIS.
• Code: All custom code can be available on request from the lead contact.
• Any additional information required to reanalyze the data reported in this paper is available from the lead contact upon request.

### Method details

#### Subjective well-being data

Individual well-being data was sourced from the China Family Panel Survey (CFPS), a nationally representative panel survey administered by the Institute of Social Science Survey at Peking University. The CFPS encapsulates 162 counties spanning across 25 provinces, accounting for 95% of mainland China's populace. Figure S1 visualizes the geographical distribution of the counties encompassed by the survey, exhibiting a higher density of counties in the eastern and central regions of the country, thereby more accurately mirroring China's population distribution.

The CFPS was inaugurated in 2010 and proceeds in a quadrennial cycle. Although supplemental surveys were carried out in inter-wave periods (e.g., in 2012 and 2016), they employed a substantially truncated survey instrument. As such, we leveraged data from three comprehensive waves, specifically those where subjective well-being queries were administered: 2010, 2014, and 2018.

To ensure that we are accurately measuring individuals’ exposure to historical temperature, we include only those who were in the same location since birth.

The following geographic information was acquired for each respondent from the CFPS:

i) Current location in each survey wave;
ii) Whether their current location is the same as at birth;
iii) Whether their location at three years old is the same as at birth;
iv) Whether their location at 12 years old is the same as at birth; and
v) The latest year in which they moved to their current location.

To accurately capture both individual and regional-level adaptation in our temperature anomalies calculation, we establish stringent inclusion criteria for respondents: they should have remained in the same locale across all survey rounds, their current location must coincide with their place of birth, and their places of residence at ages three and twelve should align with their birthplace. Additionally, the most recent year of transition to their current location should correspond with their birth year. This rigorous respondent selection methodology affords us near-comprehensive locational histories for this subset of participants, enabling the construction of precise historical reference temperatures.

Across the three CFPS waves, we accrued a total of 53,924 person-wave observations (pertaining to respondents aged 16 and above), each involving at least two interviews with the respondent. From this pool, our baseline sample is constituted of respondents who maintained their place of birth as their residence throughout. This yields a sample size of 26,583 person-wave observations, equivalent to 8,861 unique individuals.

While this method of sample selection assures us of location history, the downside is that it includes only individuals that choose to remain at the same location. As such, we show that sample selection does not affect the results (see supplemental information).

Furthermore, we execute meticulous robustness assessments employing the entire dataset, along with alternative forms of sample stratification to demonstrate that our findings are not influenced by the composition of the sample. (see Robustness checks in STAR Methods section).

The primary dependent variable used in this study is subjective well-being, which is also derived from the CFPS. Specifically, respondents were asked the following question: “*If 1 is the lowest and 5 is the highest (in CFPS 2014 and 2018, the highest is 10), how would you rate your happiness?”* To ensure comparability across all three waves, we re-scaled the measurement from CFPS 2014 and 2018 to a scale of 1-5.

While analogous metrics of self-reported happiness or subjective well-being have been utilized in extant literature to evaluate the impacts of diverse environmental amenities,^15^^,^^34^^,^^35^^,^^36^^,^^37^ the potential for misreporting or bias in self-reported measures remains a legitimate concern.

In pursuit of this objective, we clarify why subjective well-being (SWB) serves as a valid outcome in the context of this study.

First, unlike other commonly-used outcomes such as mortality or illnesses, SWB provides a general measure of one's welfare that is applicable to the entire population.^38^ For example, even if temperature increases do not lead to fatalities, they can still have adverse impacts on one's overall well-being. Nevertheless, it is essential to acknowledge that SWB is a subjective metric. To address this limitation, we leverage the longitudinal nature of CFPS and incorporate individual-level fixed effects. This approach allows us to conduct a within-respondent analysis, thereby mitigating the influence of subjectivity in the regression estimates.

Second, although SWB is inherently subjective, the respondents provided their responses devoid of explicit reference to climate change or temperature. Contrary to overt survey inquiries connected to concerns or appraisals of climate change, our approach mitigates the risk of strategic responses or attitude-behavior discrepancies frequently witnessed in climate change surveys.^39^^,^^40^

Third, even if potential misreporting of SWB by respondents exists, such misrepresentation tends to bias our regression estimates towards zero, thus leaning towards statistical insignificance.^41^

Table S1 provides a summary of the descriptive statistics for the primary variables that are deployed in the ensuing analysis. The average self-reported happiness is 4.12 (on a scale of 1 to 5) with a standard deviation of 1.03.

#### Daily weather information data

The daily weather data encompassing parameters such as temperature, wind speed, relative humidity, precipitation, and sunlight hours, were amassed from 820 weather stations throughout China for the period spanning 1951 to 2019. These datasets were obtained from the China Meteorological Data Service Center, an entity under the purview of China's National Meteorological Information Center. Given that the most granular locational information available for each respondent in the CFPS is at the county level (two administrative tiers below the province), we employ inverse-distance weighting^42^^,^^43^^,^^44^ to convert weather data from the station level to the county level.

On average, respondents experienced a temperature of approximately 23.7°C in the 30-day period leading up to the survey. Acknowledging the significant climatic heterogeneity throughout China, there exists a considerable divergence in temperature, with a standard deviation of 5.4°C. Crucially, the impacts of global warming are discernible in China, given that the mean temperature anomalies during the lifetimes of our respondents approximate to around 0.62°C. This signifies that the prevailing temperature is predominantly above the historical average.

#### Econometric model

The primary model used in this study is fixed-effects panel regression:

(Equation 1) $$y_{ijt}=\alpha +\beta_{1}f\left({temp}_{ijt}\right)+X_{ijt}\rho +\gamma_{i}+W_{ijt}\sigma +\theta_{\left(j\right)}\left(t\right)+\varepsilon_{ijt}$$

In this model, the dependent variable $y_{ijt}$ represents subjective well-being (SWB) reported by individual *i* from county *j* on interview date *t*.

To link temperature information with SWB, we utilize $f\left({temp}_{ijt}\right)$ to represent the ambient temperature in county *j* for the past 30 days. There are two ways to construct $f\left({temp}_{ijt}\right)$:

*Temperature bins*: We categorize daily average temperatures for each of the 30 days before the interview date into mutually exclusive intervals of 3°C. This approach, commonly used in climate change econometrics literature,^43^^,^^45^^,^^46^^,^^47^^,^^48^ results in $\beta_{1}$ being a vector with coefficients for each temperature bin. To ensure adequate observations in each bin, we consolidate temperatures below freezing into a single bin, given that the majority of interviews took place during summer and autumn when there are fewer instances of lower temperatures. The omitted category constitutes the 18°C-21°C bin. Hence, each coefficient can be interpreted as the impact of an additional day within its associated bin on SWB relative to the omitted bin.

*Temperature anomalies*: $f\left({temp}_{ijt}\right)$ can also be defined as temperature anomalies, which are the differences between the actual temperature and the historical reference temperature $\left({temp}_{ijt}-{hist\_temp}_{ijt}\right)$.^27^^,^^28^^,^^29^^,^^30^ The distinction from previous literature is that $\left({temp}_{ijt}-{hist\_temp}_{ijt}\right)$ varies from person to person, not just from region to region. Specifically, ${temp}_{ijt}$ is the average temperature for the 30 days before the interview date, and ${hist\_temp}_{ijt}$ represents the mean historical temperature for the respective period up until the year of the respondent's birth. For instance, the historical reference temperature for a 25-year-old respondent interviewed in end-August, 2014 is the average temperature for the month of August from 1989 to 2013. Under this setup, $\beta_{1}$ is jointly identified by variation from current temperature, and from each individual’s historical temperature exposure which in turn is driven by their respective locations, birth-dates, and survey dates.

Across both definitions of temperature, $\beta_{1}$ measures the direct impact of temperature on SWB for the following reasons.

First, there are at least three channels in which temperature can affect humans: i) direct, ii) indirect via changes to the ecosystem (e.g., increased waterborne diseases), and iii) indirect through an interaction of ecosystems and societal systems (e.g., precipitation affecting agricultural production).^49^ The second and third channels take much longer time to manifest as their effects are transmitted across complex societal systems (e.g., it will take a prolonged period of abnormal weather to affect agricultural production, and more time for the effect to be transmitted to food prices.^50^^,^^51^^,^^52^^,^^53^ On the other hand, we define temperature exposure for a relatively short period of the last 30 days.

Second, to the extent that temperature indirectly affects humans through ecosystems and societal systems, their shocks are transpired in similar fashion to everyone in the same location. In contrast, we use individualized temperature anomalies in our analyses. This means that two individuals from the same city and interviewed on the same day will most likely have different temperature anomalies.

Just as any studies that examine the impact of temperature on human outcomes, there could be potential bias in estimating $\beta_{1}$ if individuals selectively inhabit locations based on unobserved preferences. For example, Timmins^54^ found that Brazilian domestic migration patterns align with temperature differences across the country. Nevertheless, it is critical to note that locational sorting presents fewer concerns when utilizing temperature anomalies as the primary explanatory variable. This is due to the less predictable nature of temperature anomalies as opposed to absolute temperature. Additionally, individuals exhibit lesser tendencies to choose locations based on anomalous temperatures rather than the absolute temperature levels. However, there are several reasons why we can reasonably consider short-term temperature anomalies as nearly random.^11^^,^^18^

Firstly, to control for any time-invariant individual and location characteristics, we incorporate individual fixed effects $\gamma_{i}$. This level of granularity in fixed effects allows us to account for unobserved individual preferences, including locational and climate preferences. Consequently, the statistical identification of $\beta_{1}$ is based on variations at the within-respondent level.

Secondly, we incorporate interview-date fixed effects, denoted as $\left(\theta_{\left(j\right)}\left(t\right)\right)$ to comprehensively account for nationwide temporal trends or seasonality that fluctuate over time. These fixed effects are specified at the calendar date level (where 1^st^ January 2010 is a different fixed effect from 1^st^ January 2014), encompassing year, month, day-of-week, and public holidays fixed effects. Including these fixed-effects is a common practice in climate change econometric models, enabling us to interpret the temperature coefficient as causal.^11^

Thirdly, given that individual-level fixed effects solely account for time-invariant factors, we incorporate a suite of time-varying control variables into our analysis. This incorporates a vector of contemporaneous weather characteristics, $W_{ijt}$ , which include variables like hours of sunlight, precipitation, relative humidity, and wind speed, all presented up to the second order. Furthermore, we integrate $X_{ijt}$ into our model, which encompasses temporally mutable individual characteristics such as age, marital status, and educational attainment, all of which potentially hold sway over SWB.

Fourthly, compared to temperature levels, temperature anomalies exist in all regions, and regional variations in this variable are not obvious, and are not easily predicted. Using the estimation dataset, Figure S2 plots the distributions of temperature and temperature anomalies across four distinctive climatic zones in China. We can see that while there are stark differences in temperature distribution across these four zones, the disparity between their respective temperature anomalies is much less pronounced.

Lastly, the error term $\varepsilon_{ijt}$ is clustered two-way at the county and survey-date levels. The underlying assumption is that model residuals are homogeneous within region-time group, but heterogeneous between regions or even between different periods within the same region. We believe this is the most reasonable residual assumption, because the variation of the key variable, $f\left({temp}_{ijt}\right)$, exists along with these two dimensions. In a later section, we will relax several modeling assumptions for robustness checks.

#### Difference between temperature anomalies and fixed-effects model

We begin with:

(Equation 2) $$y_{ijt}=\beta_{0}+\beta_{1}{temp}_{ijt}+\mu_{ijt}$$

In Equation 2, we are examining the impact of temperature (*temp*) on outcome (*y*) of individual *i* from city *j* at time *t*.

Assuming there is more than one city in the sample, we can then include locational fixed effects $\delta_{j}$:

(Equation 3) $$y_{ijt}=\beta_{0}+\beta_{1}{temp}_{jt}+\delta_{j}+\mu_{ijt}$$

In this setup, $\delta_{j}$ controls for everything constant at the city-level, including location, land size, historical temperature, and many others. As such, $\beta_{1}$ is identified using the difference between temperature at time *t* and average temperature over the data coverage period *T*. This is because the mathematical equivalent of including city-level fixed effect is to de-mean each variable using city averages. In this regard, we can re-write Equation 3 as:

(Equation 4) $$\left(y_{ijt}-\frac{1}{T}\sum_{t=1}^{T}y_{ijt}\right)=\beta_{0}+\beta_{1}\left({temp}_{jt}-\frac{1}{T}\sum_{t=1}^{T}{temp}_{jt}\right)+\left(\delta_{j}-\frac{1}{T}\sum_{t=1}^{T}\delta_{jt}\right)+\left(\mu_{ijt}--\frac{1}{T}\sum_{t=1}^{T}\mu_{ijt}\right)$$

where $\left(\delta_{j}-\frac{1}{T}\sum_{t=1}^{T}\delta_{jt}\right)$ =0, because $\delta_{j}$ does not change over time. Equation 4 shows that following the inclusion of city-level fixed effects, $\beta_{1}$ is identified off the difference between temperature at city *j* and time *t*, and the average temperature over the data coverage period *T* for city *j*.

Toward this end, in many existing studies, temperature anomalies is defined exactly as such, i.e., the difference between temperature at city *j* and time *t*, and the average temperature of city *j*. By this definition, a locational fixed-effects temperature model is, in practice, estimating the effects of temperature anomalies.

However, the construction of temperature anomalies in our study is different as we use individual’s birth-dates and survey dates to compute a personalized historical reference temperature. Specifically, we define temperature anomalies as:

(Equation 5) $${Anomalies}_{jt\left(i\right)}={temp}_{jt\left(i\right)}-\frac{1}{Age\left(i\right)}\sum_{t\left(i\right)=birth\;year-month}^{t\left(i\right)=Interview\;year-month}{temp}_{jt\left(i\right)}$$

Under this setup, Equations 4 and 5 are identical if and only if

1) all individuals from the same city are interviewed on the same date *t*;
2) all respondents in the same city *j* have the same age; and
3) The date *t* at which all individuals from city *i* are interviewed must be after the month in which they were born (e.g., interviewed in June 2022, and born in April 1980)

While it is theoretically possible for these three conditions to be fulfilled, it is extremely unlikely in practice. As such, the estimated coefficient for temperature anomalies obtained in our study is different from the one obtained from a typical locational fixed-effects model.

Intuitively, the reason why these two models are different is because we incorporate personalized information in the construction of temperature anomalies such that two individuals (*i* and *i'*) from the same city *j*, and interviewed on the same day *t* can have different historical temperature (as long as they do not have the same birthday).

Moreover, because we are using longitudinal dataset, the same individual *i* that was interviewed in year *y* and *y’* will also have different historical temperature for each survey year if:

1) The respondent was interviewed on different dates on each survey wave (e.g., June in the first wave, and August in the second wave) and/or
2) The average temperature in the time between the first and second wave is different from time that pre-dates the surveys.

This is where even individual-level fixed effects (which we have included) will not fully absorb the effects of individualized temperature anomalies.

We also show how these two approaches are different empirically. First, we generated outcomes using simulated datasets under various data conditions, and summarize their findings in Table S5 (simulation implemented in Stata, code available upon request.). Second, we estimated variants of Equation 3 using the same dataset from the study, and show that the results are very different from the baseline findings (Table S6).

#### Robustness checks

In this section, we undertake an array of robustness tests to guarantee that the baseline findings are unmarred by factors such as sample selection or underlying modeling suppositions.

First, as our weather data only goes back to 1951, there could be imprecisions in constructing temperature anomalies for respondents born before that year. To address this concern, we include only respondents born after 1951, ensuring more accurate measurements of temperature anomalies. Even within the confines of this limited sample, the coefficient for temperature anomalies retains its statistical significance, mirroring the baseline results at -0.022 (Table S7, Column (1)).

Second, temperature covariates can be defined using various constructs, not just average temperatures. In order to explore this, we employ maximum temperature and wet-bulb temperature to construct temperature anomalies. In both instances, the coefficients for temperature anomalies are akin to those of the baseline results (Table S7, Columns (2) and (3)).

Third, besides short-term happiness, respondents also report two self-rated metrics related to their well-being: overall life satisfaction and optimism (both on a 1-5 ascending scale). As these metrics are tangentially related to SWB, we anticipate them to have similar relationships with temperature anomalies. Indeed, in both cases, temperature anomalies are negatively associated with these alternative measures of well-being, with estimated marginal effects of around -0.02 (Table S7, Columns (4) and (5)).

Fourth, the 2018 wave of the CFPS dataset only provides information on the month of the interview, not the specific date. To examine if our previous results were affected by presuming the 15^th^ day as the interview date, we experimented with an assortment of dates from the 10^th^ to the 20^th^. The coefficients for temperature anomalies largely sustained consistency with the baseline outcomes (Table S8).

Fifth, the current representation of temperature anomalies may be biased by a few instances of exceedingly abnormal temperatures, leading to imprecise estimates of its correlation with SWB. To mitigate this concern, we have redefined temperature anomalies by tallying the number of days within the same 30-day period where the daily temperature surpasses the historical average. SWB continues to exhibit a negative correlation with these day-measured temperature anomalies, with a decrease of 0.0034 in SWB for each additional day when the temperature exceeds the historical average (Table S9, Column (1)). With an average of 18.6 days, the mean effect of day-measured temperature anomalies on SWB approximated 0.06. Similar outcomes are observed when using alternative timeframes (ranging from 30 to 50 years) to construct the historical average temperature (Columns (2) to (4)).

Sixth, we currently utilize a 30-day period to establish temperature exposure. However, given the dependent variable isn't affixed to any specific timeframe, respondents' SWB may also be responsive to temperature structured using different time intervals. To investigate this, we constructed temperature anomalies using time frames varying from one week to one year. Figure S8 displays the coefficients of temperature anomalies across these varying periods. The magnitude of effects increases with the time span, ranging from -0.013 for one-week averages to -0.039 for one-year averages. However, statistical significance diminishes with longer durations. This finding suggests that the short-term impacts of ambient temperature on well-being are more immediate and accurately captured, whereas the long-term impacts entail more intricate mechanisms and are less effectively encapsulated by reduced-form models.

Seventh, although we have implemented two-way clustering of standard errors (by county and survey date) in our baseline model, alternative feasible methods for clustering standard errors exist within the dataset and the empirical approach. We tested several other clustering configurations: i) by county, ii) by county and month of survey, iii) by city, and iv) by city and month of survey. The outcomes presented in Columns (1) through (4) of Table S10 illustrate that all four specifications maintain the same level of statistical significance. This reinforces that our previous findings are not attributable solely to the choice of cluster selection.

#### Temperature projections

Projected surface temperatures for each grid point from 2015 to 2100 is obtained from the NEX-GDDP-CMIP6 dataset provided by the NASA Center for Climate Simulation in the United States. The NEX-GDDP-CMIP6 dataset comprises of bias-corrected global downscaled climate projections that are aligned with various Shared Socioeconomic Pathways (SSPs). These projections are derived from the General Circulation Model (GCM) simulations conducted within the framework of the Coupled Model Intercomparison Project Phase 6 (CMIP6).

The grid points are defined with a resolution of 0.25 degrees in latitude and 0.25 degrees in longitude. To account for model uncertainty, we employ an ensemble approach by averaging the projections from 20 climate models (see Table S11 for the full list of models). This approach allows us to mitigate the influence of individual models, and further examine the impact of model uncertainty on subjective well-being.

#### Projected impacts of temperature on subjective well-being

To conduct projections on subjective well-being, we first obtain annual temperature projections for Chinese cities from 2021 to 2100 using the IPCC-led Climate Model Intercomparison Project. As there are numerous temperature projections models, where each uses a distinctive algorithm to predict future climate, we obtain projections from 20 models, and use their averages.

These temperature projections are obtained for three distinct scenarios. SSP 585 is a “very high emissions” scenario, which depicts a future characterized by robust global economic growth, rapid urbanization, technological advancements primarily focused on fossil fuels, and limited emphasis on environmental and climate concerns. Under this scenario, greenhouse gas emissions continue to rise, with projections suggesting a global average temperature increase of more than 5.0°C by 2100 relative to pre-industrial levels, representing an extreme case in the absence of mitigation efforts.^55^

SSP 126 represents the policy scenario where governments worldwide succeed in decarbonizing their economies. Similarly, this optimistic scenario corresponds to the combination of RCP2.6 and SSP1. In here, temperature in year 2100 is projected to be around 1.7°C above pre-industrial.^55^

SSP 245 represents a middle-of-the-road scenario where decarbonatization is incomplete, and future temperature is projected to be around 2.8°C above pre-industrial.

Second, under each scenario, we compute temperature anomalies for each year in two distinct ways. First, using population projection models, we obtain the average age of Chinese citizen for each year from 2020 to 2100 (projections for age demographics are derived from the World Population Prospects as published by the United Nations. (https://population.un.org/wpp/). We then use their birth-year to compute the historical reference temperature of this representative citizen for each province. Second, in similar fashion, we compute a non-changing historical reference temperature by using the average temperature from 1980 to 2000.

Finally, we multiply the temperature anomalies for each year by the marginal impact of temperature anomalies on SWB to retrieve the impact of climate change on well-being at each year.
