# Supplementary material for: Adaptation cannot keep pace with projected temperature increase
Source: iScience. 2023 Nov 7;26(12):108403. doi: 10.1016/j.isci.2023.108403 (PMC10700836; doi:10.1016/j.isci.2023.108403)
Supplement: Document S1. Figures S1–S8 and Tables S1–S12 [file mmc1.pdf]

iScience, Volume *26*

Supplemental information

Adaptation cannot keep pace with projected temperature increase

Shuai Chen, Jie-Sheng Tan-Soo, and Hai-Jian Ye

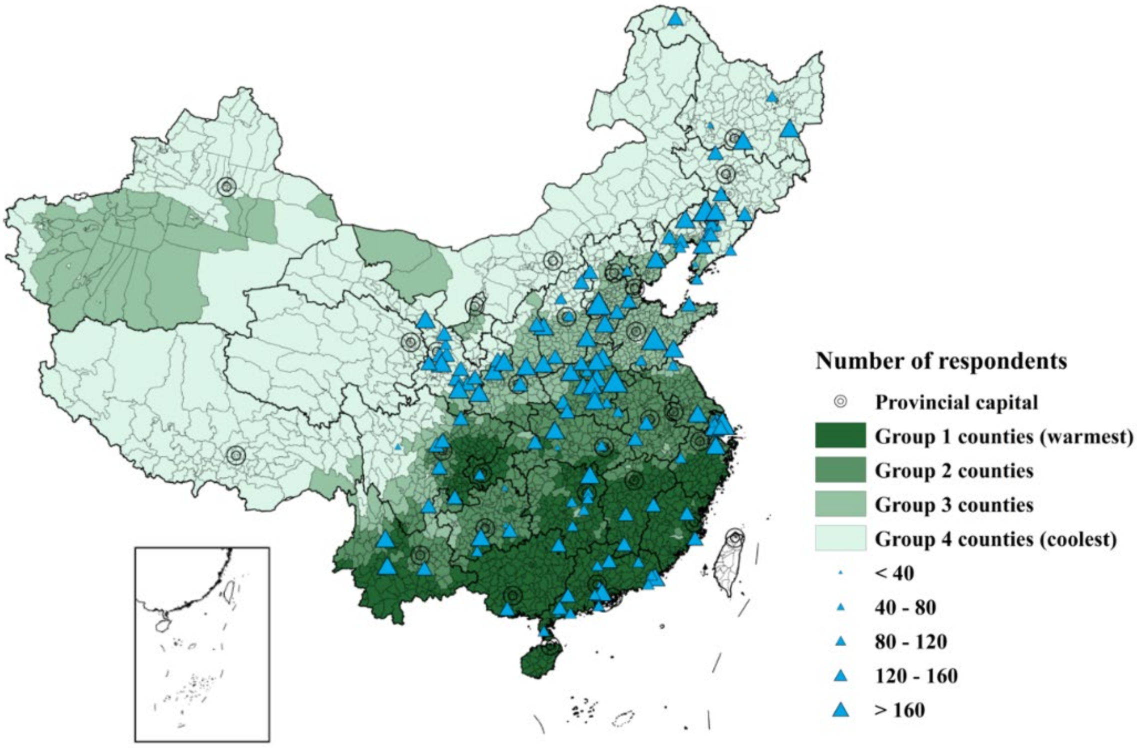

Figure S1: Geographical distribution of sample observations, related to STAR Methods. The dataset encompasses a total of 147 counties. The number of observations in each county is indicated by the size of the corresponding bubble in the analysis. We have also grouped counties according to their average annual temperature where darker shades indicate the warmer areas, and vice versa.

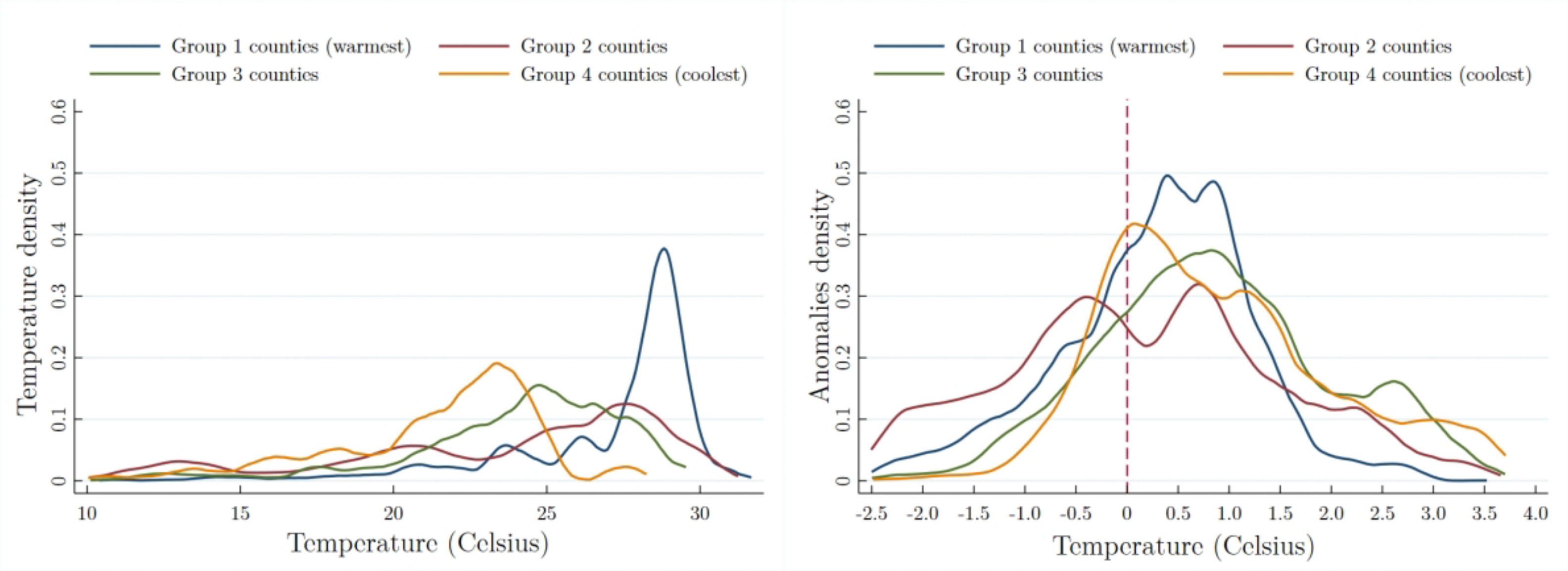

(A) (B)

Figure S2: Distribution of temperature levels and temperature anomalies by climatic zones in China, related to STAR Methods. Panel A shows distribution of current temperature from this study's dataset, and across four different climatic zones in China. Panel B shows the same for distribution of temperature anomalies.

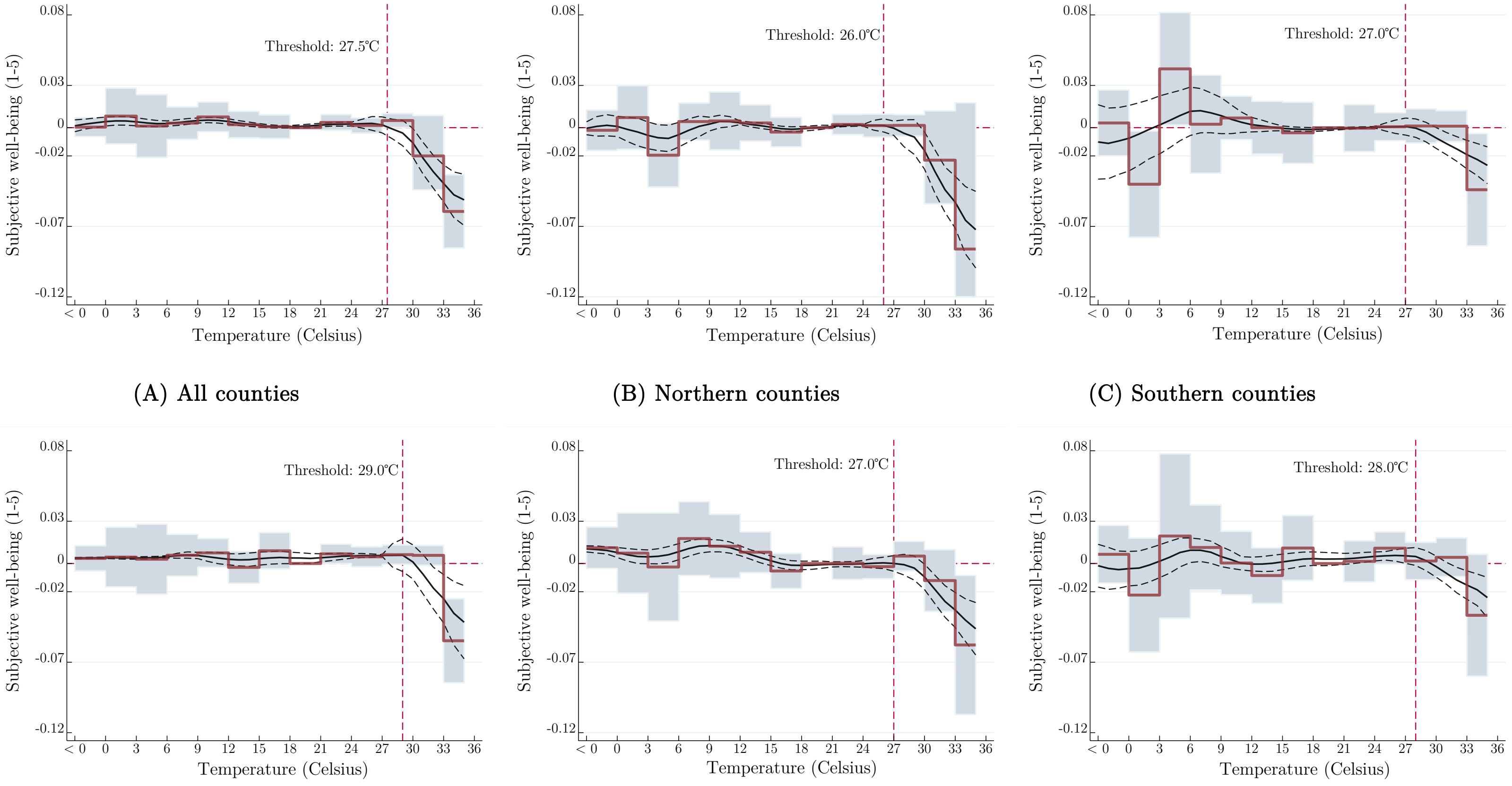

(A) All counties (B) Northern counties (C) Southern counties

(D) Eastern counties (E) Western counties (F) Central counties

Figure S3: Subjective well-being and temperature bin across different parts of China, related to Figure 1. The charts presented herein delineate the computed coefficients of temperature bins, quantified by enumerating the frequency of days within the preceding 30-day period falling within each discrete 3°C interval, as they relate to subjective well-being. Each calculated coefficient ought to be interpreted in relation to the baseline group denoted by the 18°C to 21°C temperature range. The punctuated lines depicted in each chart correspond to the 95% confidence intervals for these computed coefficients, while the vertical line signifies the threshold temperature at which the inflection point is initiated.

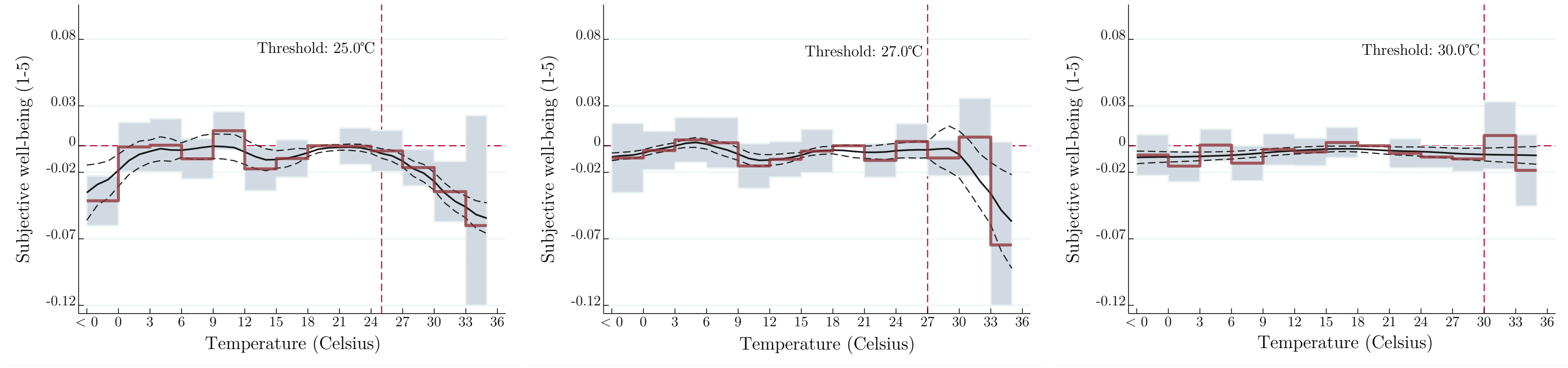

(A) 2010 (B) 2014 (C) 2018

Figure S4: Subjective well-being and temperature bin across different periods, related to Figure 1. Panel (A), (B), and (C) are estimated using data from the 2010, 2014, and 2018 CFPS respectively. The graphs here depict the estimated coefficients of temperature bins (where we count the number of days in the past 30 days belonging to each of the mutually exclusive intervals of 3°C) on subjective well-being. Each estimated coefficient should be interpreted with respect to the reference group of 18°C to 21°C. The dotted lines in each graph represents 95% confidence intervals for the estimated coefficients, while the vertical line indicates the temperature level at which the inflection point begins.

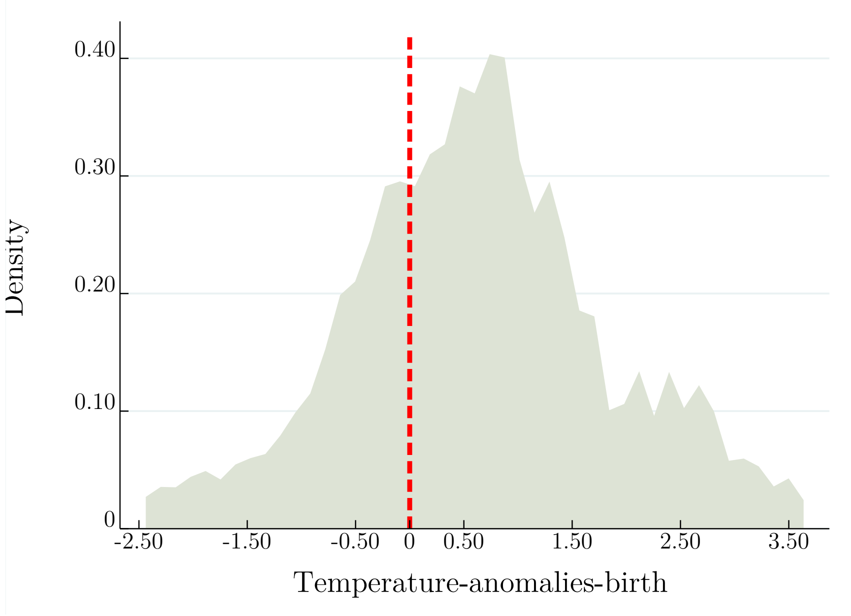

Figure S5: Histogram of temperature-anomalies-birth, related to Table 1. The presented figure illustrates the distribution of temperature-anomaly-birth values, spanning from -2.51 to 3.71, as conveyed by a histogram. The erect red line serves to denote the threshold value of 0, established to delineate the point of equilibrium between actual and historical temperatures.

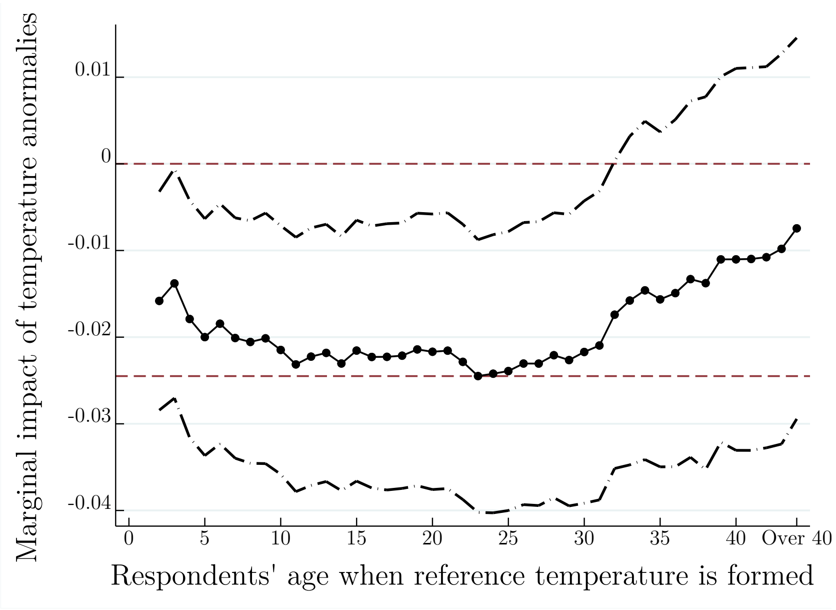

Figure S6: Formation of reference temperature at different ages, related to Table 1. The figure plots the coefficients collected from different constructions of historical reference temperature. The labels in the x-axis represent the age since birth at which historical reference temperature is averaged over.

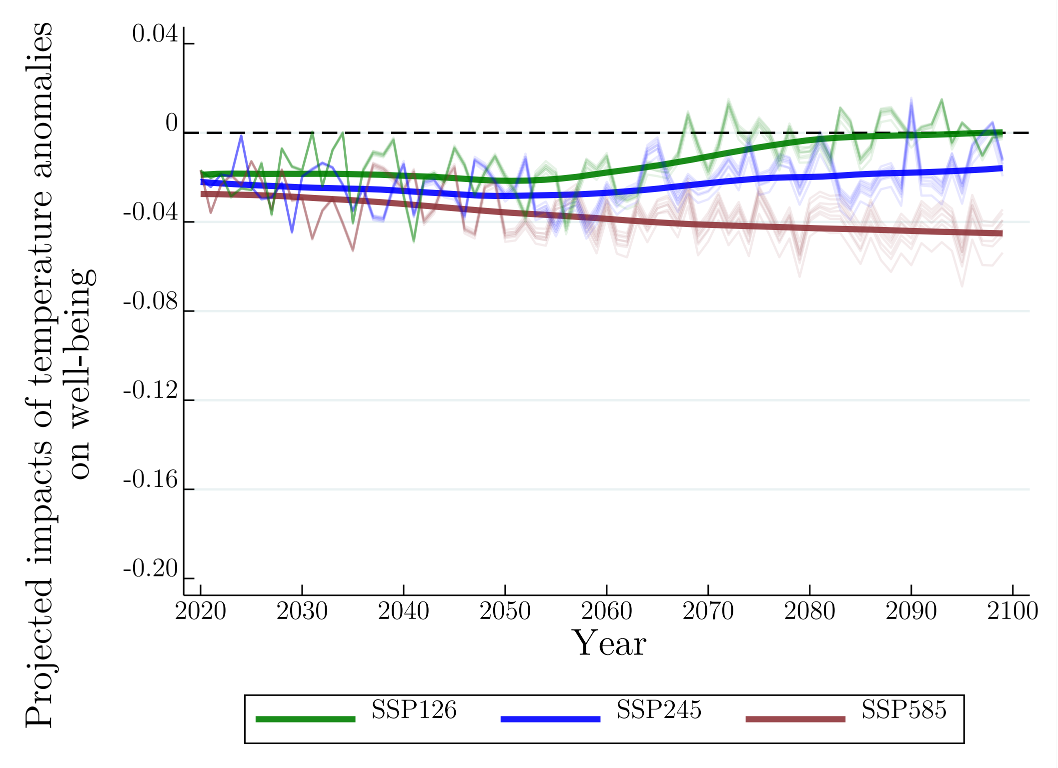

Figure S7: Projected impacts of temperature on subjective well-being, related to Figure 3. These graphs show the projected impacts of temperature on subjective well-being by computing temperature anomalies under continuously-updating according to age. Faint lines indicate province-level temperature projections. The faint lines represent different age projections.

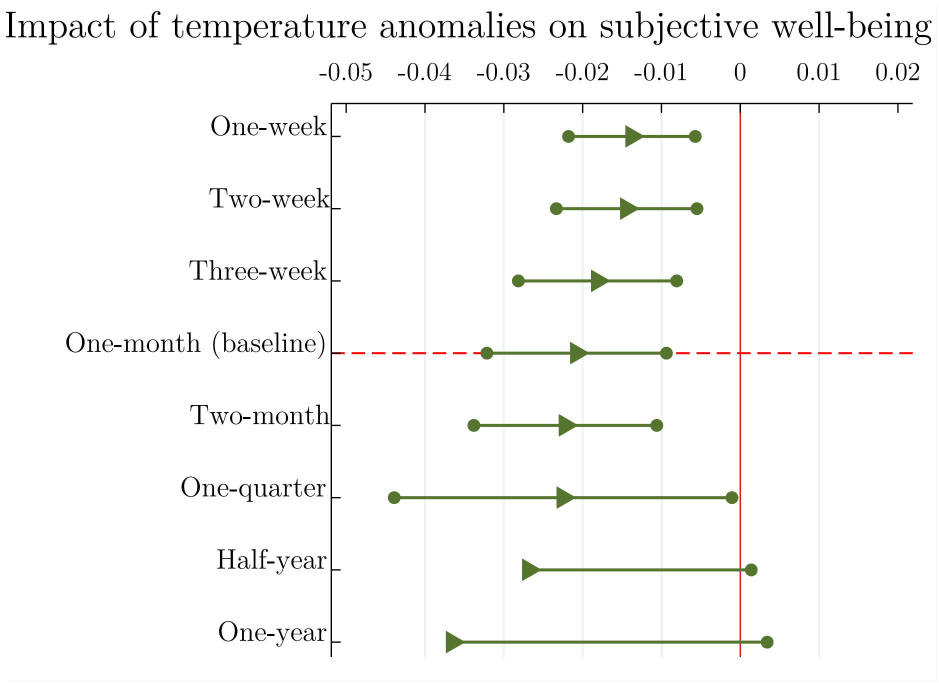

Figure S8: Different level of aggregation for historical reference temperature, related to STAR Methods. The figure represented here exhibits the estimated coefficient pertinent to temperature anomalies along with the associated 95% confidence interval, distributed across diverse aggregation timeframes.

Table S1: Descriptive statistics, related to STAR Methods.

Variable	Definition (Unit)	Mean	SD	Min	Max
Dep. Var.					
Subjective well-being	Level 1-5 (1- very unhappy; 5-very happy)	4.12	1.03	1	5
Temperature					
Current temperature	Average interview-month temperature (℃)	23.74	5.38	-24.34	31.51
Temperature-birth	Average interview-month temperature since birth-year (℃)	23.14	5.20	-22.55	29.78
Temperature-anomalies-birth	Difference between current & temperature-birth (℃)	0.62	1.18	-2.51	3.71
Weather controls					
Precipitation-birth	Average interview-month precipitation since birth-year (mm)	139.71	62.48	0.92	449.49
Solar duration-birth	Average interview-month solar duration since birth-year (hours)	200.12	32.22	35.20	274.52
Humidity-birth	Average interview-month humidity since birth-year (%)	76.01	6.41	38.46	87.80
Wind speed-birth	Average interview-month wind speed since birth-year (m/s)	2.20	0.64	0.82	5.85
Individual characteristics controls					
Age	Respondent's age on interview day	49.84	13.41	16	85
Marital status	Respondent's marital status on interview day (0: unmarried; 1: married)	0.89	0.31	0	1
Educational level	Respondent's years of schooling on interview day	5.97	4.68	0	20
Employment status	Respondent's employment status on interview day (0: unemployed; 1: employed)	0.71	0.45	0	1
Income	Per-capita household income (10,000 CNY/year) in 2010 value	1.36	2.94	0	185.83
Feeling of comfort	Feeling of discomfort in the last two weeks (0: comfortable; 1: uncomfortable)	0.33	0.47	0	1

Notes: Number of observations: 26,583. Data source: CFPS 2010, 2014, and 2018. Whenever feasible, the 'interview-month' is specified as the 30-day period prior to the interview date. In cases where the precise interview date is unspecified in the dataset, the midpoint of the interview month, specifically the 15th day, is utilized as a proxy for the interview date. Descriptive statistics corresponding to the remaining variables incorporated in other regression analyses are collated in Table S2.

Table S2: Descriptive statistics for temperature bins, related to STAR methods.

Variable	Definition (Unit)	Mean	SD	Min	Max
Temperature bins below 0	The number of days below 0℃ for each of 30 days prior to interview date	0.26	2.39	0	31
Temperature bins 0-3	The number of days between 0℃–3℃ for each of 30 days prior to interview date	0.16	1.24	0	24
Temperature bins 3-6	The number of days between 3℃–6℃ for each of 30 days prior to interview date	0.20	1.19	0	26
Temperature bins 6-9	The number of days between 6℃–9℃ for each of 30 days prior to interview date	0.32	1.45	0	19
Temperature bins 9-12	The number of days between 9℃–12℃ for each of 30 days prior to interview date	0.53	2.04	0	24
Temperature bins 12-15	The number of days between 12℃–15℃ for each of 30 days prior to interview date	0.93	2.79	0	26
Temperature bins 15-18	The number of days between 15℃–18℃ for each of 30 days prior to interview date	1.70	3.63	0	28
Temperature bins 18-21	The number of days between 18℃–21℃ for each of 30 days prior to interview date	3.27	4.78	0	26
Temperature bins 21-24	The number of days between 21℃–24℃ for each of 30 days prior to interview date	6.32	6.24	0	29
Temperature bins 24-27	The number of days between 24℃–27℃ for each of 30 days prior to interview date	8.15	6.69	0	26
Temperature bins 27-30	The number of days between 27℃–30℃ for each of 30 days prior to interview date	6.99	7.98	0	31
Temperature bins 30-33	The number of days between 30℃–33℃ for each of 30 days prior to interview date	2.15	4.11	0	25
Temperature bins over 33	The number of days over 33℃ for each of 30 days prior to interview date	0.03	0.39	0	7

Notes: Number of observations: 26,583. Data source: CFPS 2010, 2014, and 2018. Whenever feasible, the 'interview-month' is specified as the 30-day period prior to the interview date. In cases where the precise interview date is unspecified in the dataset, the midpoint of the interview month, specifically the 15th day, is utilized as a proxy for the interview date.

Table S3: Construction of historical reference temperature using different samples, related to STAR methods.

Dep. Var.	Subjective well-being (level 1-5)						
	(1)	(2)	(3)	(4)	(5)	(6)	(7)
Temperature-anomalies -birth	-0.0216**	-0.0208***	-0.0218***	-0.0213***	-0.0232***	-0.0227***	-0.0240***
	(0.0098)	(0.0069)	(0.0069)	(0.0069)	(0.0078)	(0.0087)	(0.0084)
Scenario	Full sample	Same location in 2010, 2014, 2018	(2)+Location at three years old same as birth-place	(3)+Location at 12 years old same as birth-place	(4)+Location at birth same as current	(5)+Never left birth-place	(6)+Left birth-place
Observations	53,924	39,112	38,133	36,771	30,703	26,583	27,324

Notes: All models are estimated with individual-level fixed effects, calendar date fixed effects, and survey weights. Column (1) is additionally estimated with county-level fixed effects. Weather controls are relative humidity, wind speed, hours of sunlight, and precipitation, and included up until second order polynomials. Individual controls are per-capita household income, age, marital status, educational level, and feeling of discomfort in the last two weeks. Sample selection is based on locational history information, and Column (6) is baseline result. All results in this study can be replicated using the samples in Column (2) and (7), and are available upon request. Standard errors are listed in parentheses and clustered at county- and date-level (two-way clustering). *** $p<0.01$, ** $p<0.05$, * $p<0.1$.

Table S4: Historical temperature constructed at different milestones, related to Table 1.

Dep. Var.	Subjective well-being (level 1-5)				
	(1)	(2)	(3)	(4)	(5)
Temperature-anomalies -birth	-0.0160**	-0.0189***	-0.0175**	-0.0249**	-0.0076
	(0.0071)	(0.0057)	(0.0076)	(0.0115)	(0.0121)
Scenarios	Infant (0 - 3)	Childhood (3 - 11)	Teenager (11 - 18)	Young adult (18 - 40)	Adult (after 40)
Date *FE*	Yes	Yes	Yes	Yes	Yes
Individual *FE*	Yes	Yes	Yes	Yes	Yes
Weather controls	Yes	Yes	Yes	Yes	Yes
Individual controls	Yes	Yes	Yes	Yes	Yes
Observations	26,601	26,533	26,539	18,989	14,990

Notes: All models are estimated with survey weights, and include the following set of covariates: survey dates fixed-effects (FE), individual-level FE, weather controls, and individual controls. Weather controls are relative humidity, wind speed, hours of sunlight, and precipitation, and included up until second order polynomials. Individual controls are per-capita household income, age, marital status, educational level, and feeling of discomfort in the last two weeks. Standard errors are listed in parentheses and clustered at county- and date-level (two-way clustering). *** p<0.01, ** p<0.05, * p<0.1.

Table S5: Empirical comparison between individualized temperature anomalies and locational fixed-effects model using simulated datasets, related to STAR Methods.

Dataset characteristics	*Multiple locations*	Yes						
	Survey month	Identical	Different	Different	Different	Identical	Different	Different
	Survey year	Identical	Identical	Identical	Identical	Identical	Different	Different
	Birth month	Identical	Identical	Identical	Different	Different	Different	Identical
	Birth year	Identical	Identical	Identical	Different	Different	Different	Identical
	Difference between coefficients for temperature and temperature anomalies	Both coefficients cannot be identified	Both coefficients are identical if all survey months are after birth month	Both coefficients are different as long as some survey months are before birth months	Both coefficients are different	Coefficient for temperature cannot be identified whereas it can be for temperature anomalies	Both coefficients are different	Both coefficients are different

Notes: All models are estimated with locational fixed-effects using data simulated on Stata. Code available upon request.

Table S6: Empirical comparison between individualized temperature anomalies and locational fixed-effects model using study's dataset, related to STAR Methods.

Dep. Var.	Subjective well-being (level 1-5)		
	(1)	(2)	(3)
Temperature-anomalies-birth	-0.0227***		
	(0.0087)		
Current temperature		0.0048	-0.0054
		(0.0045)	(0.0048)
Date *FE*	Yes	Yes	Yes
Individual *FE*	Yes	No	Yes
County *FE*	No	Yes	No
Observations	26,583	26,583	26,583

Notes: All models are estimated with survey weights. Weather controls are relative humidity, wind speed, hours of sunlight, and precipitation, and included up until second order polynomials. Individual controls are per-capita household income, age, marital status, educational level, and feeling of discomfort in the last two weeks. Column (1) is baseline findings, and included for comparison. Standard errors are listed in parentheses and clustered at county- and date-level (two-way clustering). *** $p<0.01$, ** $p<0.05$, * $p<0.1$.

Table S7: Robustness checks, related to STAR Methods.

Dep. Var.	Subjective well-being (level 1-5)			Life satisfaction (level 1-5)	Optimism (level 1-5)
	(1)	(2)	(3)	(4)	(5)
Temperature-anomalies-birth	-0.0215***	-0.0210**	-0.0200**	-0.0246***	-0.0222***
	(0.0080)	(0.0091)	(0.0078)	(0.0093)	(0.0071)
Scenarios	Born after 1951	Daily max. temperature	Wet-bulb temperature	Alternate dependent variable	
Date *FE*	Yes	Yes	Yes	Yes	Yes
Individual *FE*	Yes	Yes	Yes	Yes	Yes
Weather controls	Yes	Yes	Yes	Yes	Yes
Individual controls	Yes	Yes	Yes	Yes	Yes
Observations	19,383	26,583	26,583	26,583	26,583
Mean [SD] of *Dep. Var.*		4.12 [1.03]		3.80 [1.04]	3.92 [1.04]

Notes: All models are estimated with survey weights, also include the following set of covariates: survey dates fixed-effects (FE), individual-level FE, weather controls, and individual controls. Weather controls are relative humidity, wind speed, hours of sunlight, and precipitation, and included up until second order polynomials. Individual controls are per-capita household income, age, marital status, educational level, and feeling of discomfort in the last two weeks. Standard errors are listed in parentheses and clustered at county- and date-level (two-way clustering). *** $p<0.01$, ** $p<0.05$, * $p<0.1$.

Table S8: Assumption of different survey dates, related to STAR Methods.

Dep. Var.	Subjective well-being (level 1-5)									
	(1)	(2)	(3)	(4)	(5)	(6)	(7)	(8)	(9)	(10)
Temperature-anomalies	-0.0226***	-0.0225***	-0.0226***	-0.0222***	-0.0226***	-0.0222**	-0.0216**	-0.0220**	-0.0230**	-0.0244**
-birth	(0.0083)	(0.0083)	(0.0083)	(0.0085)	(0.0086)	(0.0088)	(0.0090)	(0.0090)	(0.0091)	(0.0093)
Scenarios	10th	11th	12th	13th	14th	16th	17th	18th	19th	20th
Observations	26,507	26,516	26,527	26,553	26,551	26,583	26,543	26,493	26,470	26,470

Notes: The 2018 wave of CFPS only include survey month and not exact date. In the baseline, we assumed the survey was implemented on the 15th of the month for the 2018 round. In this robustness check, we relax this assumption and used other dates. All models are estimated with survey weights, also include the following set of covariates: survey dates fixed-effects (FE), individual-level FE, weather controls, and individual controls. Weather controls are relative humidity, wind speed, hours of sunlight, and precipitation, and included up until second order polynomials. Individual controls are per-capita household income, age, marital status, educational level, and feeling of discomfort in the last two weeks. Standard errors are listed in parentheses and clustered at county- and date-level (two-way clustering). *** p<0.01, ** p<0.05, * p<0.1.

Table S9: Day-measured temperature anomalies, related to STAR Methods.

Dep. Var.	Subjective well-being (level 1-5)			
	(1)	(2)	(3)	(4)
Temperature-anomalies-day	-0.0034***	-0.0033**	-0.0036**	-0.0037**
(# of days average daily temperature exceed the historical average)	(0.0012)	(0.0014)	(0.0015)	(0.0015)
Reference period for historical average temperature	since respondent's birth year	previous 30 years	previous 40 years	previous 50 years
Mean [S.D.] of Temperature-anomalies-day	18.60 [6.10]	20.23 [6.70]	20.52 [6.39]	20.66 [6.13]

Notes: Number of observations: 26,583. All models are estimated with survey weights, also include the following set of covariates: survey dates fixed-effects (FE), individual-level FE, weather controls, and individual controls. Weather controls are relative humidity, wind speed, hours of sunlight, and precipitation, and included up until second order polynomials. Individual controls are per-capita household income, age, marital status, educational level, and feeling of discomfort in the last two weeks. Standard errors are listed in parentheses and clustered at county- and date-level (two-way clustering). *** $p<0.01$, ** $p<0.05$, * $p<0.1$.

Table S10: Robustness checks for modeling assumptions, related to STAR Methods.

Dep. Var.	Subjective well-being (level 1-5)			
	(1)	(2)	(3)	(4)
Temperature-anomalies-birth	-0.0227**	-0.0227***	-0.0227**	-0.0227***
	(0.0097)	(0.0079)	(0.0094)	(0.0075)
Scenarios	Cluster (county)	Cluster (county and year-of-month)	Cluster (city)	Cluster (city and year-of-month)
Date *FE*	Yes	Yes	Yes	Yes
Individual *FE*	Yes	Yes	Yes	Yes
Weather controls	Yes	Yes	Yes	Yes
Individual controls	Yes	Yes	Yes	Yes
Province-by-year *FE*	No	No	No	No

Notes: Number of observations: 26,583. Weather controls are relative humidity, wind speed, hours of sunlight, and precipitation, and included up until second order polynomials. Individual controls are per-capita household income, age, marital status, educational level, and feeling of discomfort in the last two weeks. Standard errors are listed in parentheses and clustered at county- and date-level (two-way clustering). *** $p<0.01$, ** $p<0.05$, * $p<0.1$.

Table S11: CMIP6 temperature projection models used in study, related to Figure 3.

SSP126	SSP245	SSP585
ACCESS-CM2	ACCESS-CM2	ACCESS-CM2
ACCESS-ESM1-5	ACCESS-ESM1-5	ACCESS-ESM1-5
AWI-CM-1-1-MR	AWI-CM-1-1-MR	AWI-CM-1-1-MR
BCC-CSM2-MR	BCC-CSM2-MR	BCC-CSM2-MR
CIESM	CIESM	GFDL-ESM4
CMCC-ESM2	CMCC-ESM2	CMCC-ESM2
CanESM5	CanESM5	CanESM5
EC-Earth3	EC-Earth3	EC-Earth3
FIO-ESM-2-0	FIO-ESM-2-0	INM-CM4-8
MIROC6	MIROC6	MIROC6
MPI-ESM1-2-HR	MPI-ESM1-2-HR	MPI-ESM1-2-HR
MPI-ESM1-2-LR	MPI-ESM1-2-LR	MPI-ESM1-2-LR
MRI-ESM2-0	MRI-ESM2-0	MRI-ESM2-0
NESM3	NESM3	INM-CM5-0
INM-CM4-8	INM-CM4-8	INM-CM4-8
FGOALS-g3	FGOALS-g3	FGOALS-g3
GFDL-ESM4	GFDL-ESM4	GFDL-ESM4
IPSL-CM6A-LR	IPSL-CM6A-LR	IPSL-CM6A-LR
MRI-ESM2-0	MRI-ESM2-0	MRI-ESM2-0
TaiESM1	TaiESM1	TaiESM1

Notes: Near-surface temperature for each grid point from 2015 to 2100, is obtained from the NEX-GDDP-CMIP6 dataset. This dataset is provided by the NASA Center for Climate Simulation in the United States.

Table S12: Project impact difference between continuously-updating and fixed reference, related to Figure 3.

	Period	2020s	2030s	2040s	2050s	2060s	2070s	2080s	2090s
Project impact in SSP126	Static reference temp.	-0.0284	-0.0221	-0.0425	-0.0517	-0.0528	-0.0438	-0.0413	-0.0423
	Dynamic reference temp.	-0.0207	-0.0131	-0.0218	-0.0250	-0.0162	-0.0042	-0.0015	-0.0001
	Gap	-0.0077	-0.0091	-0.0207	-0.0267	-0.0366	-0.0396	-0.0399	-0.0422
Project impact in SSP245	Static reference temp.	-0.0331	-0.0359	-0.0469	-0.0657	-0.0695	-0.0709	-0.0860	-0.0855
	Dynamic reference temp.	-0.0235	-0.0252	-0.0241	-0.0334	-0.0237	-0.0191	-0.0217	-0.0128
	Gap	-0.0096	-0.0106	-0.0228	-0.0324	-0.0458	-0.0517	-0.0643	-0.0727
Project impact in SSP585	Static reference temp.	-0.0473	-0.0567	-0.0801	-0.0906	-0.0882	-0.1115	-0.1231	-0.1384
	Dynamic reference temp.	-0.0319	-0.0334	-0.0472	-0.0475	-0.0280	-0.0429	-0.0403	-0.0406
	Gap	-0.0154	-0.0232	-0.0329	-0.0431	-0.0602	-0.0686	-0.0828	-0.0978

Notes: This table shows the difference in temperature anomalies between Figure 4 panel (A) and Figure 4 (B) at each decade.
